# Mechanical Performance of Thoracic Aortic Stent-Grafts: An In Vitro and In Silico Study

**DOI:** 10.1007/s10439-025-03949-y

**Published:** 2025-12-21

**Authors:** Anna Ramella, Sara Barati, Giulia De Campo, Giulia Luraghi, Jose Felix Rodriguez Matas, Frederic Heim, Nabil Chakfé, Tim J. Mandigers, Irene Fulgheri, Maurizio Domanin, Santi Trimarchi, Francesco Migliavacca

**Affiliations:** 1 Department of Chemistry, Materials and Chemical Engineering “G. Natta”, Politecnico di Milano, Leonardo da Vinci, 20133 Milano, Italy; 2 AllStent srl, Milan, Italy; 3 GEPROMED, Strasbourg, France; 4 Laboratoire de Physique et Mecanique des Textiles, Université de Haute-Alsace, Mulhouse, France; 5 Department of Vascular Surgery, Kidney Transplantation and Innovation, Strasbourg University Hospitals, Strasbourg, France; 6 Department of Cardiothoracic Surgery, St. Antonius Hospital, Nieuwegein, The Netherlands; 7 Fondazione IRCCS Ca’ Granda Ospedale Maggiore Policlinico, Milan, Italy; 8 Department of Clinical Sciences and Community Health, Università degli Studi di Milano, Milan, Italy

**Keywords:** TEVAR, FEA, Validation, Stent-graft, Mechanical performance

## Abstract

Thoracic endovascular aortic repair (TEVAR) is the standard of care for thoracic aortic pathologies, and its clinical success is related to the choice of stent-grafts (SGs). In this study, we conducted a comprehensive assessment of four commercial SGs (Valiant Captivia (VC), Terumo RelayPro Bare Stent (TBS), Cook Zenith Alpha (CZA), and Gore CTAG (CTAG)) to evaluate their mechanical performance in idealised and patient-specific conditions. High-fidelity finite element models were developed and validated against experimental tests and in vitro TEVAR procedures in 3D-printed rigid phantoms. The validation showed strong agreement between simulations and experiments (average error < 5%).

Then, the SGs were virtually deployed in two aortic models to investigate device-wall interaction through geometrical and mechanical parameters. A greater metal density led to increased graft apposition (up to 94%) and increased radial forces (up to 354 N vs 116N). Conversely, sparser metal structures produced lower but more localised stress regions: maximum values of 0.25 MPa versus 0.49 MPa with denser metal. Higher stresses may contribute to improved device fixation and, when associated with greater apposition, may reduce the risk of endoleak. Nevertheless, high stresses could potentially induce long-term vascular remodelling.

These results underscore the influence of SG’s design on TEVAR outcomes and support the integration of validated computational simulations into pre-operative planning. The SG performance varied across patient anatomies: this study highlights the importance of personalized device selection and establishes a foundation for using in silico methods to optimize TEVAR strategies and mitigate procedural risks.

**Supplementary Information:**

The online version contains supplementary material available at 10.1007/s10439-025-03949-y.

## Introduction

Thoracic endovascular aortic repair (TEVAR) has become the primary treatment option for pathologies involving the thoracic aorta and the aortic arch, due to its enhanced perioperative and short-term outcomes compared to open surgery [8]. Because of the complex aortic morphology, achieving proper apposition and fixation of the stent-graft (SG) onto the aortic wall may be associated with a significant challenge [1, 16]. Effective SG implantation demands adequate proximal and distal aortic landing zones and robust anchoring to securely exclude the diseased aortic segment from blood circulation without damaging the vessel wall [9] and to prevent complications such as SG migration and/or type I endoleak. Consequently, the outcome of the TEVAR procedure is related to the accurate choice of the device design and size in relation to the anatomy of the vessel. In this regard, Prendes et al. [20] studied the optimal device oversizing range with respect to the vessel when planning TEVAR. Also, in a study comparing the performance of different commercial SG, Canaud et al. [5] investigated the behaviour of proximal fixation by deploying the devices in cadaveric aortas, changing the aortic neck angulation.

Recently, computational models have been widely adopted in the literature to replicate in silico SG implantation procedures in thoracic and abdominal aortas. They can support pre-operative planning and give insights into the mechanical behaviour of the anatomical structures, devices, or their interactions [3, 17]. In this context, some research groups have used in silico methods to evaluate the mechanical behaviour of SG, both under idealised testing conditions and when implanted in patient-specific anatomies. Demanget et al. [10, 11] studied the flexibility of commercial abdominal SG by replicating bending tests with numerical simulations. In 2018, Ma et al. [15] investigated the effect of the increased thoracic SG length and oversizing on the stress exerted on the aortic wall within a patient-specific aortic model. Similarly, in 2024, Kan et al. [13] evaluated the biomechanical implications of three commonly used stent designs for TEVAR in terms of aorta morphological changes and stress distribution in a patient-specific aortic dissected model. In 2019, Hemmler et al. [12] investigated the effect of changing the abdominal stent-graft geometry by studying parameters related to potential complications.

Despite these studies, the literature is still lacking a comprehensive validation of both numerical thoracic SG models and TEVAR simulations, following standards or guidelines [2, 18, 19]. Therefore, this paper aims to provide validation evidence for four commercially available SGs commonly used during TEVAR by comparing numerical and experimental data. Then, the mechanical performance of the SG and the influence of the device design on the TEVAR procedural outcome are investigated by simulating SG implantations in two patient-specific anatomies using the Finite Element analysis (FEA).

## Materials and Methods

### Stent-Graft Models

**Fig. 1:**
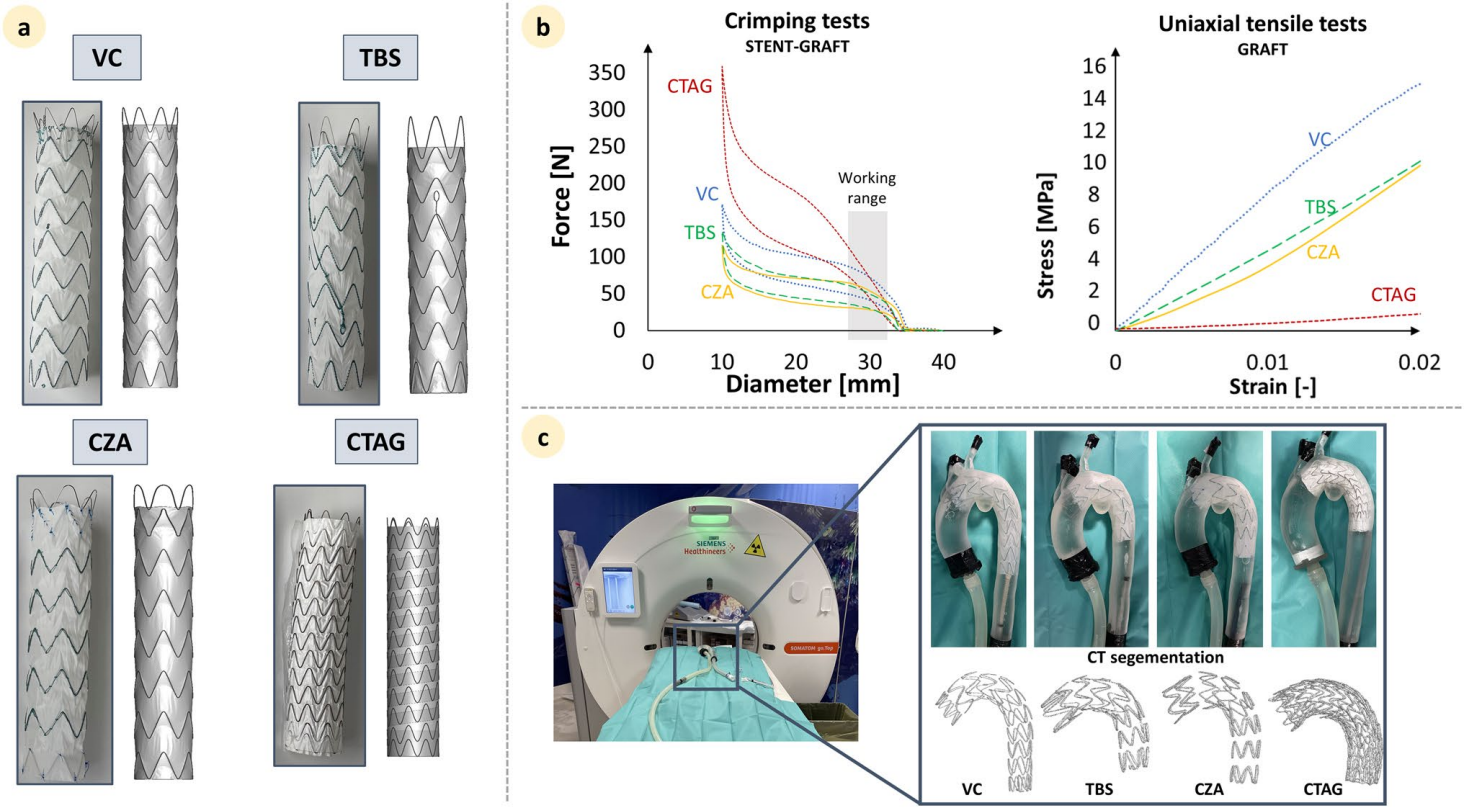
**a** The real stent-grafts and the numerical counterpart with stent ring prestress (when present). **b** Stent-graft crimping and graft uniaxial tensile test experimental curves. The overall stent-graft working range (27 mm–32 mm) is represented by the grey rectangle. **c** Experimental set-up for the validation of the TEVAR simulation and stent-graft segmentation from CT images. VC: Valiant Captivia; TBS: Terumo RelayPro Bare Stent; CZA: Cook Zenith Alpha; CTAG: Gore CTAG

Four commercially available SG (Fig. 1a) were included in the study: Valiant Thoracic SG with Captivia delivery system (*VC*) (Medtronic Vascular, Santa Rosa, CA, USA) [24], Terumo RelayPro Bare Stent stent-graft (*TBS*) (Terumo Aortic, Vascutek Ltd, United Kingdom) [25], Cook Zenith Alpha thoracic stent-graft (*CZA*) (Cook Inc., Bloomington, IN, USA) [26], and Gore C-Tag stent-graft (CTAG) (W.L. Gore & Associates, Flagstaff, AZ, USA) [27]. The first three are composed of multiple nitinol stent rings between layers of polyester (PET) graft. Stent rings have a sinusoidal shape; they are sutured to the graft, and the proximal end is an open bare stent segment. The number and position of suture points between the stent and graft change amongst different models, with CZA having the lowest number of sutures. Differently, CTAG consists of an expanded polytetrafluoroethylene (ePTFE) graft supported over its entire length by a single wire nitinol frame (stent) with a sinusoidal shape. The stent is attached to the external surface of the graft by bonding tape, and the proximal end of the device consists of an open bare stent.

VC, TBS and CZA are also characterized by a stent ring prestress [7, 22]. In fact, when removing suture points which link the stent to the graft, the nitinol rings relaxed to a larger diameter (stress-free diameter increase) with respect to the graft, in different percentages according to the device. Table 1 reports some details of the devices used in this study in terms of diameters, total length, covered graft length, working range (i.e., range of inner intended aortic vessel diameter), stent and graft thicknesses and metal-to-artery (MTA) ratio. The MTA ratio for the whole device was defined as the area of the stent in contact with a cylinder placed around the device. It was calculated as half of the lateral area of the metal stent struts (${A}_{lateral - stent}$) over the lateral area of the cylinder (${A}_{lateral-cylinder}$):

**Table 1:** Details of the four stent-grafts: reported data are from actual measurements on the devices

SG model	Proximal × distal diameter [mm]	Total SG length (covered length) [mm]	SG working range [mm]	Stent thickness [mm]	Graft thickness [mm]	MTA ratio (MTA ratio proximal 1 cm)	Stent stress-free diameter increase [%]
VC	34 × 34	178 (167)	From 29 to 31	0.50 (0.25 for the proximal spring)	0.1	11.2% (20%)	From 7.5% to 17.5%
TBS	34 × 34	160 (142)	From 30 to 31	0.50	0.115	10.9% (21.1%)	From 16.5% to 58%
CZA	34 × 34	173 (161)	30	0.50	0.115	8.2% (14.6%)	From 1.5% to 10%
CTAG	34 × 34	138 (133)	From 27 to 32	0.50	0.11	19.9% (22.6%)	N/A

SG, stent-graft; VC, Valiant Captivia; TBS, Terumo RelayPro Bare Stent; CZA, Cook Zenith Alpha; CTAG, Gore CTAG; MTA, Metal-to-Artery.

$$MTA ratio=\left(\frac{\frac{{A}_{latera{l}_{-}stent}}{2}}{{A}_{latera{l}_{cylinder}}}\right)*100$$

### Stent-Graft Numerical Models

SG geometrical models were recreated in Solidworks (Dassault Systèmes SOLIDWORKS Corp., MA, USA) or using ad hoc Python codes and then discretised in ANSA Pre-Processor v24.0.1 (BETA CAE System, Switzerland). In all the models, the stent was discretised with 2-node (linear) beam elements with Hughes–Liu formulation and the graft with 3-node membrane elements [22]. Average element sizes and number of nodes and elements for the stent and graft are listed in Table 2. They have been selected after a grid sensitivity analysis as in [22].

**Table 2.** Stent-graft mesh details in terms of the number of elements in the graft and stent components

SG model	Graft average element size	# Graft elements	Stent average element size	# Stent elements
VC	1 mm	21615	1 mm	1743
TBS	1 mm	20255	1 mm	1923
CZA	1 mm	20139	1 mm	1680
CTAG	0.75 mm	33089	1 mm	2888

SG, stent-graft; VC, Valiant Captivia; TBS, Terumo RelayPro Bare Stent; CZA, Cook Zenith Alpha; CTAG, Gore CTAG

The presence of the suture points (VC, TBS, and CZA) or the bonding tape (CTAG) was considered by creating a node-to-node connection between the stent and the graft parts. Also, the prestress state of the stent struts, when present, was modelled following the same FEA approach proposed by Ramella et al. [22].

All FEA simulations were carried out on 28 CPUs of an Intel Xeon64 with 250 GB of RAM using the commercial explicit finite element solver LS-DYNA 971 Release 14.0 (ANSYS, Inc., Canonsburg, PA, USA).

### Validation of Device Models and TEVAR Simulations

The calibration of device material properties and the validation of SG numerical models and TEVAR simulations were carried out by comparing numerical simulations with experimental tests, following the framework of our previous work [22].

#### Experimental Crimping and Uniaxial Tensile Testing

Crimping and release experimental tests were performed on single stent struts and whole device models at 37 °C ± 2 °C dry air using the Blockwise Crimper system (Blockwise Engineering LLC, AZ, USA) [22]. Starting from the initial configuration, each component was crimped down to 5 mm (for stent struts tests) or 10 mm (for whole device tests) and then released back to the initial diameter. Radial force-versus-diameter history curves were obtained (Fig. 1b). They were used to calibrate and validate the nitinol material parameters and device models. For the CTAG, it was not possible to perform single stent strut crimping tests because the nitinol frame is composed of a single sinusoidal wire. Nitinol parameters were therefore calibrated by matching the experimental and numerical curves for the whole device. Figure 1b shows the crimping curves for whole device tests.

Regarding the graft, uniaxial tensile tests under displacement control until rupture were carried out on graft samples (0.02 mm/s rate, with Bose EnduraTEC 3200 – Bose Corporation, MN, USA). All tested materials revealed a linear elastic behaviour in tension (Fig. 1b-right). The resultant Young’s modulus in the longitudinal direction was computed by linearizing the initial portion of the stress–strain curve up to ε = 0.015, following the approach adopted in our previous work (Table 3).

**Table 3:** Material parameters resulting from the graft uniaxial tensile tests and the stent nitinol parameters calibration process

GRAFT		VC [22]	TBS	CZA	CTAG
		PET	PET	PET	ePTFE
Young's Modulus	${{\boldsymbol{E}}}_{\boldsymbol{ }}$**[MPa]**	1060	520	520	90

STENT		VC [22]	TBS	CZA	CTAG
Austenite Young’s Modulus	${{\boldsymbol{E}}}_{{\boldsymbol{A}}}$**[GPa]**	57.5	57.5	57.5	34.5
Austenite Poisson’s Ratio	${{\boldsymbol{\nu}}}_{{\boldsymbol{A}}}$**[-]**	0.3	0.3	0.3	0.3
Martensite Young’s Modulus	${{\boldsymbol{E}}}_{{\boldsymbol{M}}}$**[GPa]**	47.8	47.8	47.8	29.8
Martensite Poisson’s Ratio	${{\boldsymbol{\nu}}}_{{\boldsymbol{M}}}$**[-]**	0.3	0.3	0.3	0.3
Transformation Strain	${\boldsymbol{\varepsilon}}$**[-]**	0.063	0.063	0.063	0.063
Start Transformation Loading	${{\boldsymbol{\sigma}}}_{{\boldsymbol{S}}}^{{\boldsymbol{L}}}$**[MPa]**	550	550	550	500
End Transformation Loading	${{\boldsymbol{\sigma}}}_{{\boldsymbol{E}}}^{{\boldsymbol{L}}}$**[MPa]**	620	620	620	800
Start Transformation Unloading	${{\boldsymbol{\sigma}}}_{{\boldsymbol{S}}}^{{\boldsymbol{U}}}$**[MPa]**	450	350	350	400
End Transformation Unloading	${{\boldsymbol{\sigma}}}_{{\boldsymbol{E}}}^{{\boldsymbol{U}}}$**[MPa]**	250	270	270	200
Start transformation stress in compression	$\boldsymbol{\alpha }$**[-]**	0.0279	0.0279	0.0279	0.0279

PET, Polyethylene terephthalate; ePTFE, Expanded polytetrafluoroethylene; VC, Valiant Captivia; TBS, Terumo RelayPro Bare Stent; CZA, Cook Zenith Alpha; CTAG, Gore CTAG.

#### Material Calibration and Device Validation

Crimping simulations were set up following Ramella et al. [22] by placing 12 crimping rigid planes around the device and displacing them radially to reach the target diameter. Soft penalty-based contacts were defined between the stent and each plane. The force-diameter curves obtained in the simulation were compared with the experimental ones. The calibrated nitinol material parameters are reported in Table 3.

#### Validation of Stent-Graft Implantation Simulations

##### Experimental TEVAR in Rigid Phantoms

In order to validate the release steps for each SG, a patient-specific aortic model was segmented using VMTK (Orobix s.r.l.) from anonymised Computed Tomography (CT) images provided by IRCCS Cà Granda Ospedale Policlinico di Milano. The aorta was 3D-printed with a rigid transparent resin with a uniform thickness of 1.8 mm, closed and filled with water at 37 °C to reproduce the body’s physiological temperature. Under a CT scan, a vascular surgeon deployed each prosthesis and CT acquisitions were performed at the end of deployment (Fig. 1c). Then, the stent configuration was segmented from CT images.

##### Numerical TEVAR Simulation in Rigid Phantoms

As in the experiment, the aorta was modelled with a rigid material. It was discretised with triangular shell elements with an average element size of 1 mm (75431 elements and 37839 nodes) with ANSA Pre-Processor v24.0.1 (BETA CAE System, Switzerland).

Each device has its own deployment sequence; therefore, ad hoc FE simulations were set up. For VC, TBS, and CZA, the implantation simulation followed the steps of the “*tracking method*” proposed by Ramella et al. [22, 23]: the device was crimped and displaced inside the aorta (tracking phase) until the proximal landing zone was reached and then gradually deployed. On the other hand, the deployment simulation for CTAG included the following steps: (i) crimping into a first catheter; (ii) tracking of the first catheter up to the proximal landing zone; (iii) gradual release of the SG into a second catheter proximally towards distally, and (iv) complete release into the aorta from the distal to the proximal SG region. Videos of the simulation of SGs’ deployments are reported in the supplementary material.

Penalty-based contacts with soft formulation were defined between the SG and the cylinder/catheters and between the device and the aorta [4].

The TEVAR simulation outcome was validated by overlapping the stent simulated with the one segmented from CT images. The opening area (OA) at each stent strut, both for simulations and CTs, was computed, and the percentage error (OA% error) between the two was quantified. Cut planes were considered for the CTAG, as no single stent struts were present [22].

### Stent-Graft Implantation in Compliant Phantoms

#### Numerical Simulation in Patient-Specific Anatomies

In order to compare the performance of the different SG models in realistic scenarios, each SG was virtually implanted in two patient-specific deformable anatomies (ID01 and ID02). SGs were modelled with the validated material properties; stent prestress was included when present. Patient-specific aortic models were segmented from anonymised CT images provided by IRCCS Cà Granda Ospedale Policlinico di Milano using VTMK (Orobix s.r.l.). Both aortic models were discretised in ANSA Pre-Processor v24.0.1 (BETA CAE System, Switzerland) with 3-node shell elements and an average element size of 1 mm (75431 elements for ID 1 and 68471 elements for ID 2). A thickness of 1.8 mm and linear elastic material with Young’s modulus of 2 MPa was assigned to the aortic wall [4, 21, 23]. The aortic prestress was included using Ansys Mechanical (ANSYS, Canonsburg, PA, USA) [21], considering a diastolic pressure of 80 mmHg.

#### Numerical Simulations in Idealised Anatomies

To study solely the influence of the device design, TEVAR procedure outcome was evaluated under idealised conditions, with the SG deployed inside a straight compliant tube with a diameter of 30 mm and material properties as those assigned to the aorta and discussed in the paragraph above.

#### Post-Processing

The performance of the stent-grafts is assessed in terms of:

I. *Graft-to-wall apposition (G-APP)*. A distance map was generated for each device to qualitatively assess graft proximity to the aortic wall (apposition-non-apposition map). Quantitatively, G-APP was defined as the percentage of the graft surface with a distance lower than 1 mm from the aortic wall. G-APP was calculated along the full graft length (i.e., aortic covered length) and at 1 mm-spaced cross-sections from the proximal rim to the distal end of the graft. Maximum and average values in the proximal and distal 1 cm are computed as well. Also, in order to study solely the influence of the device design on this parameter, G-APP was evaluated under idealised conditions.
II. *Stress on the aortic wall*. The von Mises stress distribution in the aortic wall was computed before and after device implantation.
III. *Contact forces (CF), net friction forces (NFF) and stent apposition density (SAD)*. CF were assessed at the end of deployment, with the aorta segmented into 2 cm regions. CF frequency distributions were extracted for each region. NFF*,* indicating device resistance to migration (e.g., pull-out), was derived from CF by scaling it with a friction coefficient of 0.1. Additionally, SAD quantified the number of aortic wall nodes per region with non-zero friction force.

## Results

### Validation of Device Models and TEVAR Simulations

By analysing the experimental crimping curve, all devices showed the typical hysteretic behaviour of shape memory alloys. Different mechanical responses can be observed amongst devices. At the maximum crimping diameter (10 mm), the CTAG device exhibited the greatest force (359.4 N), followed by the VC (171.8 N), TBS (135.3 N), and CZA (116.1 N). Analysing the working range of these SG (i.e., unloading path between 28 and 32 mm), VC, TBS, and CZA SG showed an almost constant force curve (ΔF of 15N for VC, 13N for TBS, and 5N for CZA) whilst it varied for the CTAG, ranging from 65N at 28 mm to 20N at 32 mm (ΔF of 45N).

**Fig. 2:**
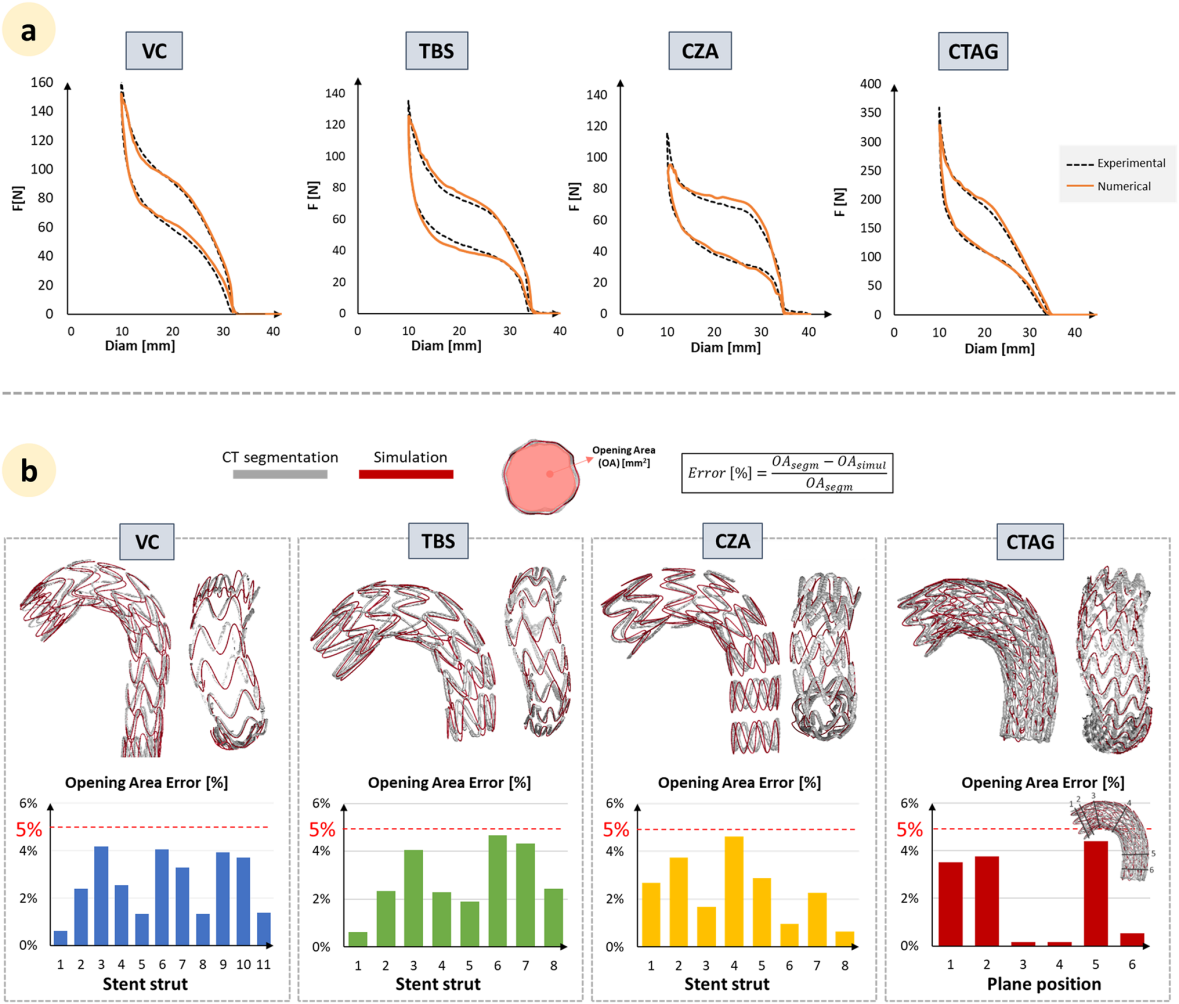
**a** Force-diameter curves for the experimental tests (black, dotted line) and the numerical simulations (orange, continuous line). **b** Comparison between deformed configurations obtained from Computed Tomography reconstructions and numerical simulations after stent-graft deployments. Histograms report the percentage difference in each stent strut or along selected planes between experiments and simulations. VC: Valiant Captivia; TBS: Terumo RelayPro Bare Stent; CZA: Cook Zenith Alpha; CTAG: Gore CTAG

Figure 2a shows the results of the device model validation in the numerical simulation (orange, continuous line). The crimping curve is able to replicate the experimental results (black, dotted line) for all device models (R^2^ = 0.8 for VC, R^2^ = 0.8 for TBS, R^2^ = 0.6 for CZA, and R^2^ = 0.8 for CTAG), especially in the unloading path.

The TEVAR simulation was validated by comparing the simulation results with the stent segmented from the CT image. Results are reported in Fig. 2b: a good SG positioning and shape matching was achieved for all SG models. OA% errors were lower than 5% in all cases (on average 2.8% ± 1.5%).

### TEVAR Simulation in Patient-Specific Anatomies

#### Simulation Results

**Fig. 3:**
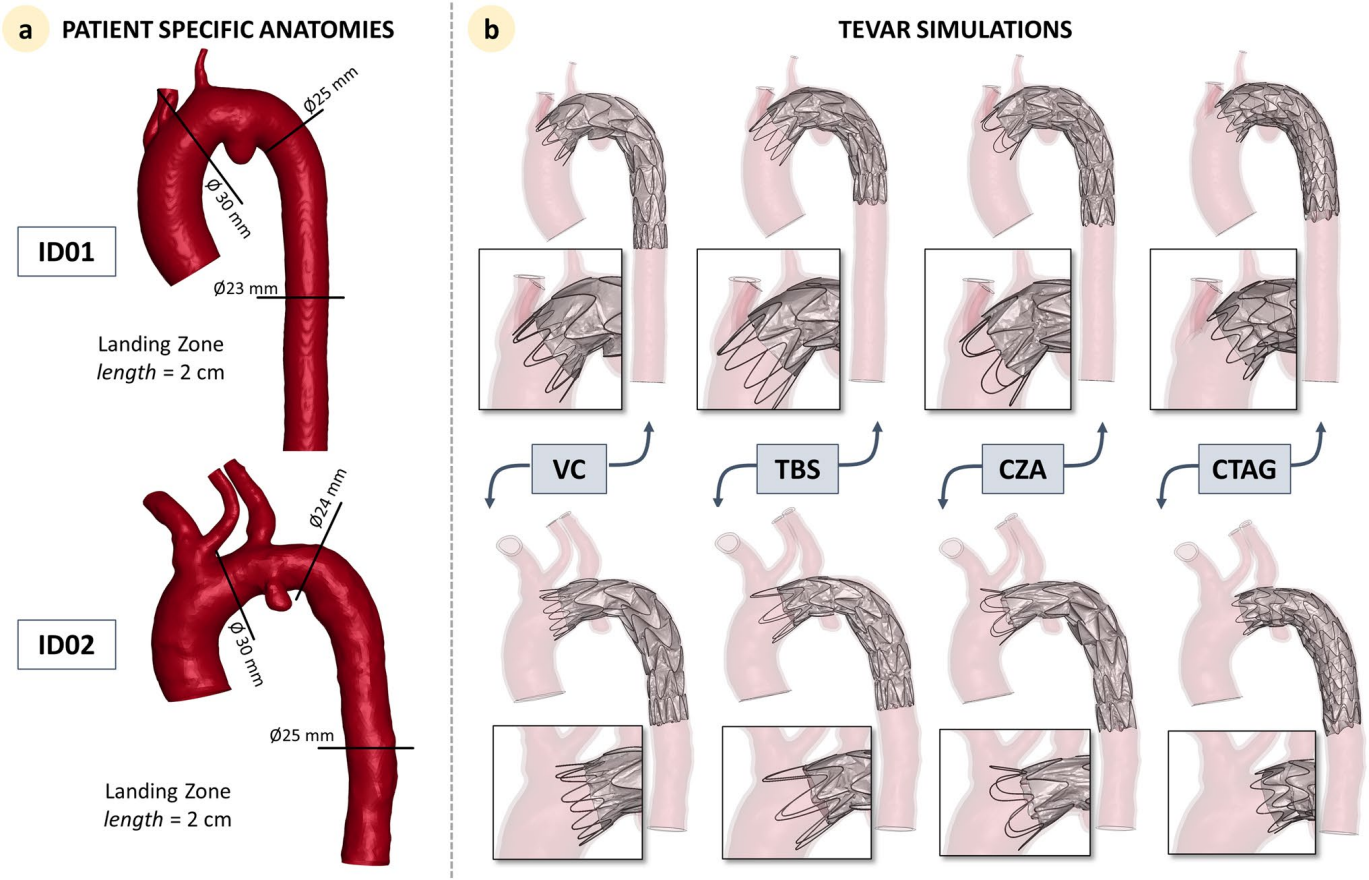
**a** Patient-specific anatomies with indications of diameters at relevant sections and **b** final stent-grafts configuration after the computational implantation for all the devices. In the small boxes, a zoom on the proximal landing zone is reported. VC: Valiant Captivia; TBS: Terumo RelayPro Bare Stent; CZA: Cook Zenith Alpha; CTAG: Gore CTAG

Figure 3a depicts the two patient-specific anatomies. The SG landing zone in the simulations was selected based on the device’s instructions for use (IFU) and after discussion with clinicians. The final deployed configuration is reported in Fig. 3b. As shown in the figure, the starting point of the graft-covered region in the sealing zone varied amongst the devices. According to the IFUs, the proximal edge of the SG in the VC, TBS, and CZA devices should be positioned at the origin of the supra-aortic branches, with the proximal uncovered stent (free flow) deployed over the vessels’ origin. In contrast, the CTAG device has proximally short bare stent struts, called apexes that are intended as part of the regular sealing zone that should not be deployed over the vessels’ origin. As a consequence, the covered portion of the sealing zone is shorter for CTAG. Details on this are reported in the boxes in Fig. 3b. for each patient and device.

#### Graft Apposition

**Fig. 4:**
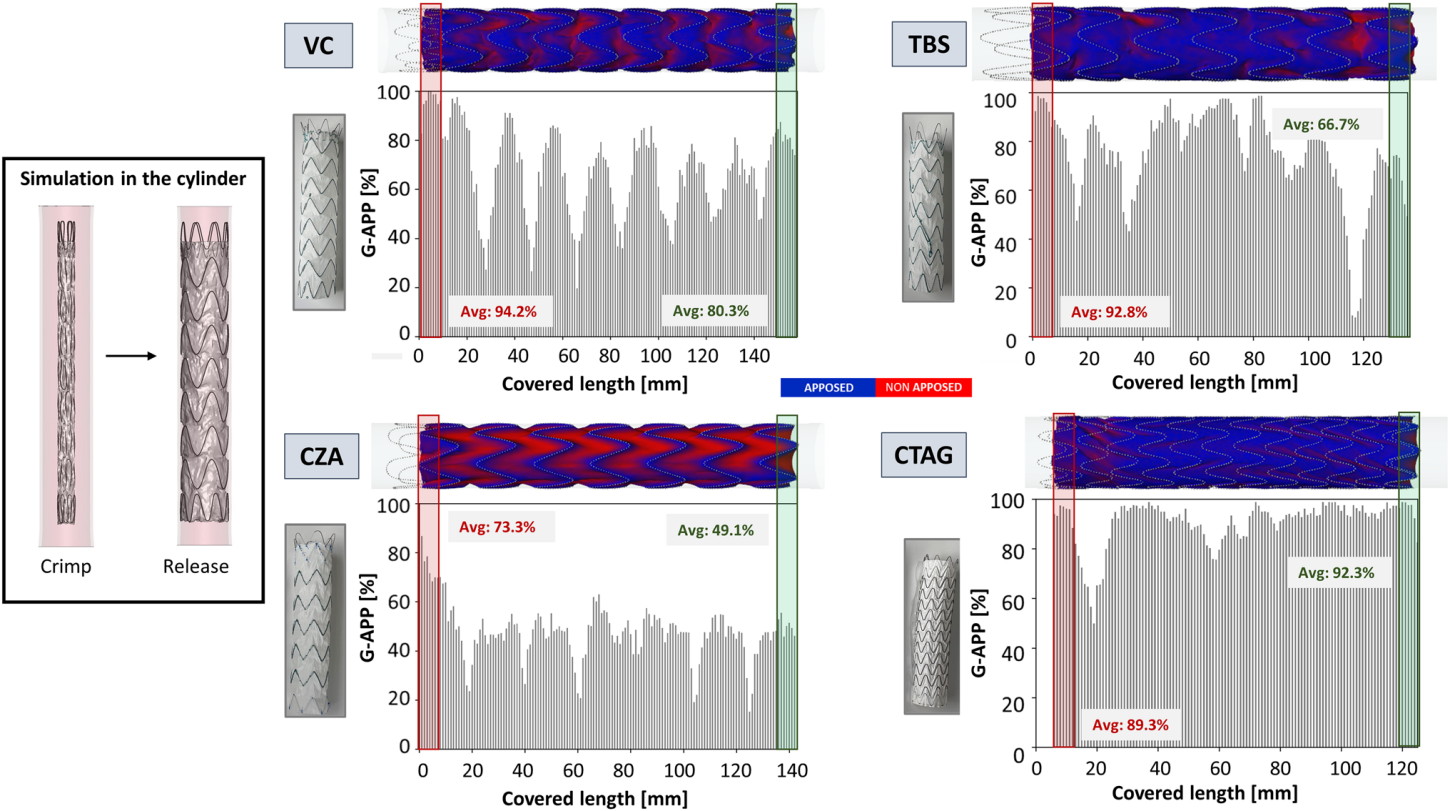
On the left, crimping and release simulation in the cylinder. On the right, apposition contour map and graft apposition (G-APP) plots for the idealised cylindrical cases. For the plots, in red is highlighted the proximal landing zone, and in green the distal one. Average G-APP percentage values in the proximal (red) and distal (green) regions are reported. VC: Valiant Captivia; TBS: Terumo RelayPro Bare Stent; CZA: Cook Zenith Alpha; CTAG: Gore CTAG. Considering the different CTAG’s IFU with respect to the other, as pointed out in paragraph 5.2.2, the plot of the G-APP values for CTAG is shifted by 5 mm towards the right, where 5 mm is the height of the bare stent

Figure 4 presents the results of the idealised cylindrical scenario through an apposition contour map, G-APP plots at 1-mm intervals along the covered length (i.e., graft length), and average G-APP values in the proximal and distal 1 cm segments. In the contour map, regions where the SG lies within 1 mm of the wall are shown in blue, whilst red areas indicate low apposition, typically due to graft folding. Considering the different CTAG IFU with respect to the other pointed out in paragraph 5.2.1., the plot of the G-APP values for CTAG is shifted by 5 mm towards the right, where 5 mm is the height of the bare stent. In any case, the quantification of the G-APP values in the proximal 1 cm follows its definition and starts from the proximal rim of the graft.

Table 4 reports the average and maximum G-APP in the idealised condition over the covered length and proximal and distal 1 cm.

**Table 4.** Average (Avg) and maximum (Max) graft apposition (G-APP) G-APP values in the idealised condition for the covered length, proximal and distal 1 cm

Device	Avg G-APP covered length (%)	Avg (Max) G-APP - proximal 1 cm	Avg (Max) G-APP - distal 1 cm
VC	67.8	94.2% (100%)	80.3% (87.4%)
TBS	75.2	92.8% (98.6%)	66.7% (74.7%)
CZA	47.8	73.3% (86.8%)	49.1% (55.6%)
CTAG	89.6	89.3% (97.5%)	92.3% (98.7%)

VC, Valiant Captivia; TBS, Terumo RelayPro Bare Stent; CZA, Cook Zenith Alpha; CTAG, Gore CTAG.

**Fig. 5:**
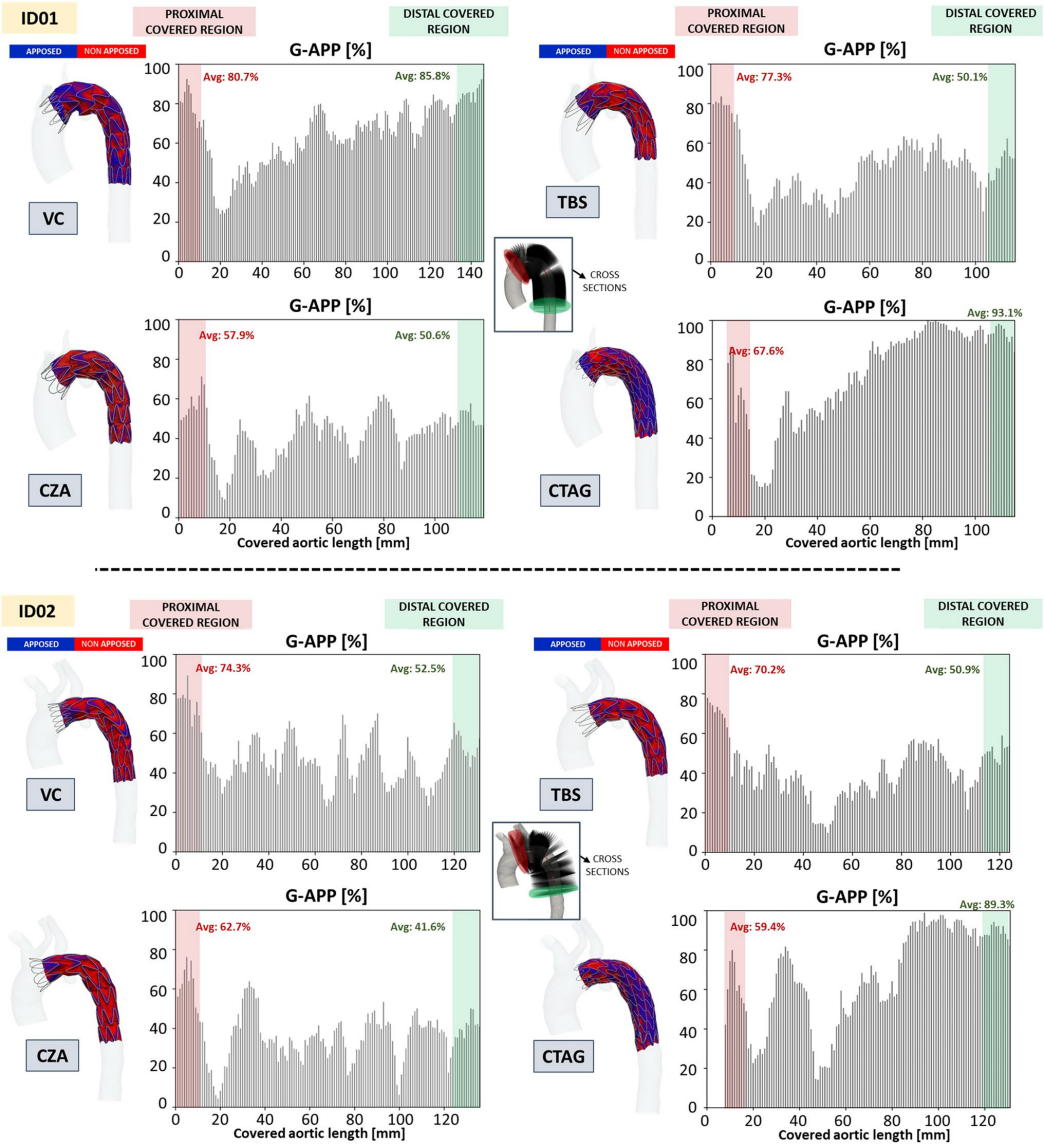
Apposition contour map and graft apposition (G-APP) plots for all scenarios. In red is highlighted the proximal landing zone, and in green the distal one. In the middle, the position of the planes for each patient. VC: Valiant Captivia; TBS: Terumo RelayPro Bare Stent; CZA: Cook Zenith Alpha; CTAG: Gore CTAG

Figure 5 illustrates the same variables as for Fig. 4, the two patient-specific cases.

In the two patients, G-APP quantification is performed on the graft-covered region. As already mentioned for the cylinder, the starting point for G-APP extraction is consistently set at the same anatomical landmark across all devices—specifically, the origin of the LCCA branch, corresponding to landing zone 2. Considering the IFU for CTAG, the first 5 mm of the sealing zone are uncovered because of the presence of the bare apexes, which is why the G-APP values are not shown in the plots.

Amongst all devices, CTAG exhibited the highest average G-APP coverage across the entire graft-covered length, with values of 71.1% for ID01 and 65.2% for ID02. VC followed, showing average values of 64.4% for ID01 and 47.7% for ID02. In contrast, TBS and CZA demonstrated slightly lower coverage, with TBS of 47.2% and 41.2%, and CZA of 42.8% and 37.3% for ID01 and ID02, respectively.

Table 5 reports the average G-APP and proximal/distal G-APP values for the two patients and for each stent-graft, as well as the maximum reached values in the proximal and distal 1-cm.

**Table 5.** For both patients, average (Avg) and maximum (Max) graft apposition (G-APP) values for the covered length, proximal and distal 1 cm (G-APP-1) and proximal and distal covered landing zone portion (G-APP-LZ)

	Device	Avg G-APP covered length (%)	Avg (Max) G-APP – proximal 1 cm	Avg (Max) G-APP – distal 1 cm
Patient ID01	VC	64.4	80.7% (92.5%)	85.8% (96.8%)
	TBS	47.2	77.3% (83.5%)	50.1% (62.3%)
	CZA	42.8	57.9% (71.3%)	50.6% (54.2%)
	CTAG	71.1	67.6% (86.4%)	93.1% (98.2%)
Patient ID02	VC	47.7	74.3% (89.3%)	52.5% (65.4%)
	TBS	41.2	70.2% (77.9%)	50.9% (58.9%)
	CZA	37.3	62.7% (76.2%)	41.6% (50.1%)
	CTAG	65.2	59.4% (79.9%)	89.3% (94.3%)

VC, Valiant Captivia; TBS, Terumo RelayPro Bare Stent; CZA, Cook Zenith Alpha; CTAG, Gore CTAG.

**Fig. 6:**
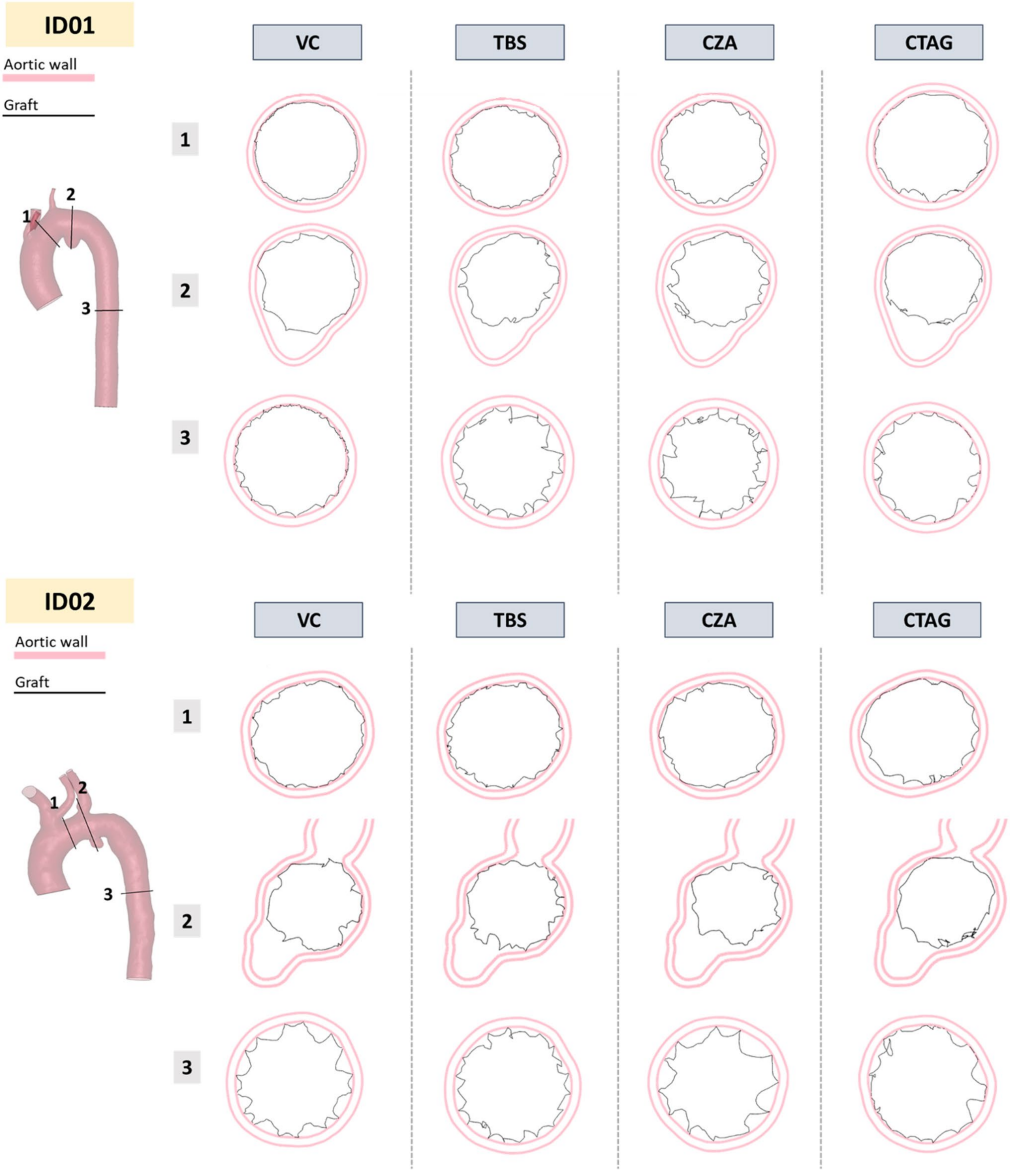
Cross-sections at proximal graft rim (1), pathology (2), and distal landing zone (3) levels. In pink is the aortic wall thickness, in black the graft. VC: Valiant Captivia; TBS: Terumo RelayPro Bare Stent; CZA: Cook Zenith Alpha; CTAG: Gore CTAG

Figure 6 shows the proximal (1), pathological (2), and distal (3) cross-sections to highlight the presence of foldings of the graft. Foldings increase moving from proximal to the pathological and distal areas because of changes in the aortic shape and diameters.

#### Stress on the Aortic Wall

**Fig. 7:**
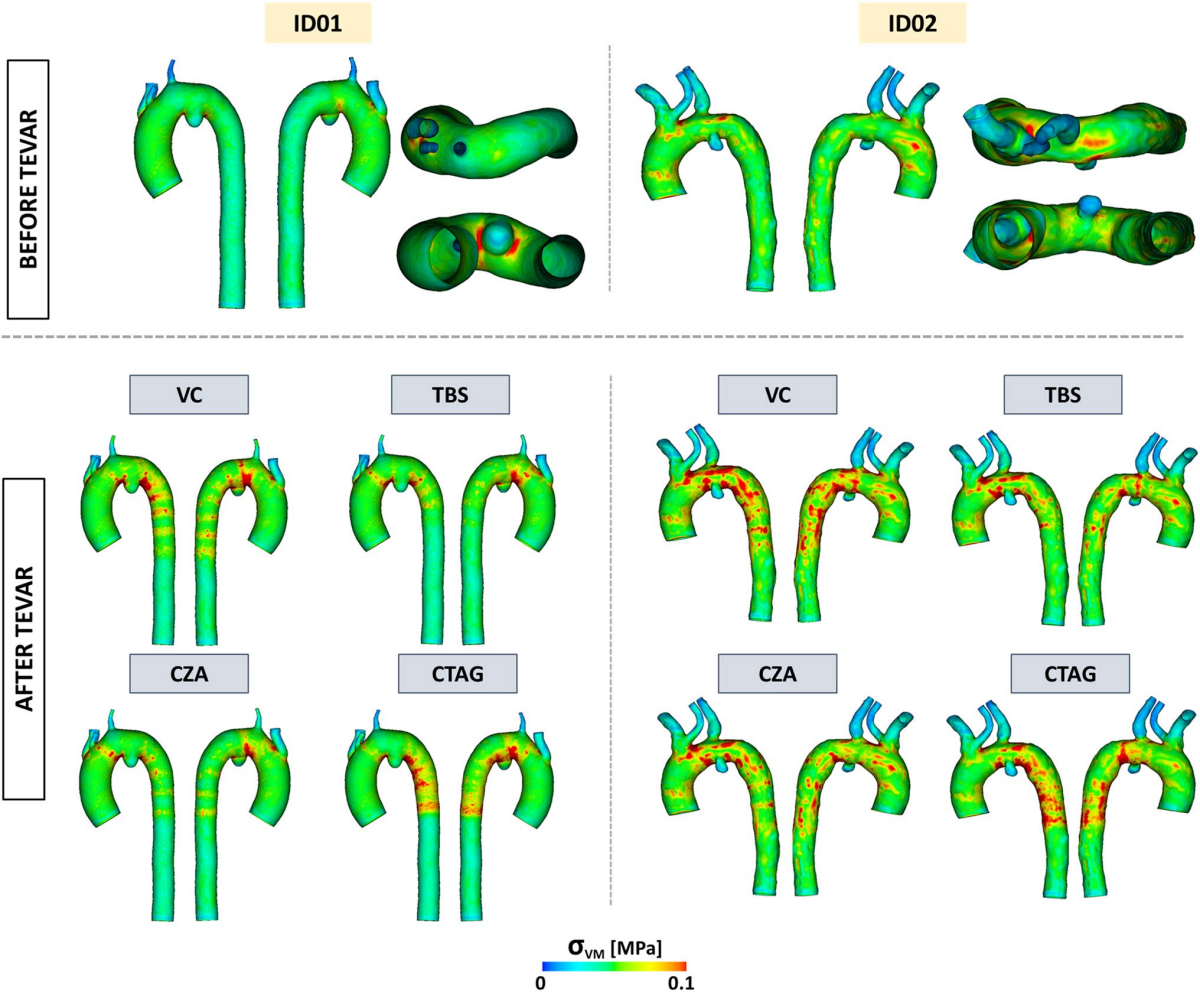
Von Mises (VM) stress distribution on the two patients before (top) and after (bottom) the TEVAR procedure. VC: Valiant Captivia; TBS: Terumo RelayPro Bare Stent; CZA: Cook Zenith Alpha; CTAG: Gore CTAG

Before the implantation, the average von Mises stress on the entire anatomy due to the vessel prestress due to diastolic pressure of 80 mmHg was 0.039 MPa for ID01 and 0.045 MPa for ID02, with maximum values of 0.33 MPa and 0.32 MPa for ID01 and ID02, respectively. In both patients, higher stress values occurred at the origin of the supra-aortic branch and at regions of narrow curvature near the Penetrating Aortic Ulceration (PAU) (Fig. 7).

After TEVAR, there was a global increase in both average and maximum von Mises stresses due to the effect of device expansion. For all devices, considering the aortic arch, the location of the maximum stress region remained unchanged from the pre-operative condition.

#### Contact and friction forces distribution

**Fig. 8:**
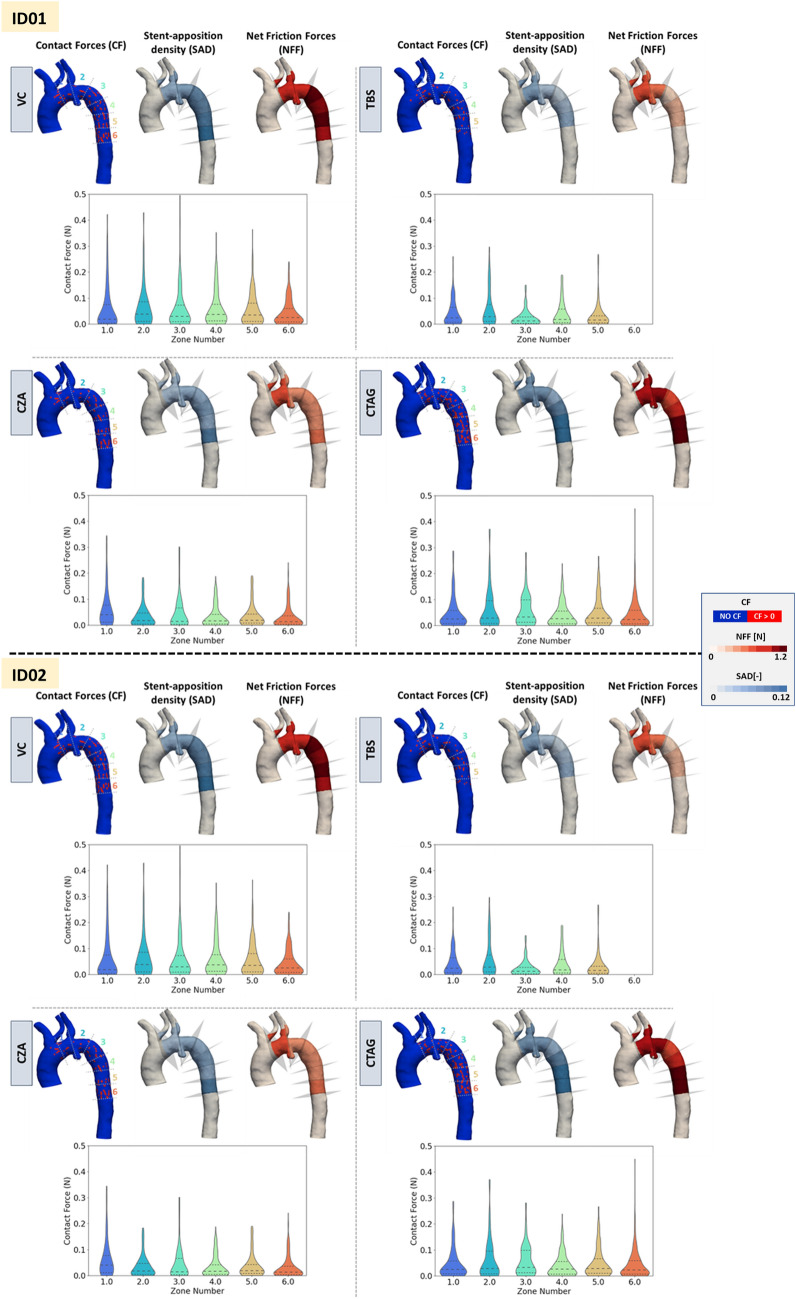
Contact Forces (CF) contour plot, Net Friction Forces (NFF) and Stent Apposition Density (SAD) distribution for patient ID01 (top) and ID02 (bottom). The Contact force distribution violin plots are reported at the bottom for each device. Different colours represent the different regions. The numbers in the CF contour plot represent the region ID. VC: Valiant Captivia; TBS: Terumo RelayPro Bare Stent; CZA: Cook Zenith Alpha; CTAG: Gore CTAG

Figure 8 shows the contact forces (CF) contour, CF frequency plots, the stent apposition density (SAD), and net friction forces (NFF) distributions. In both anatomies, looking at the CF contour plot, the amount of non-zero CF regions in the vessel proximal and distal regions was higher than in the region of the pathology or narrow curvature. Regarding the analysis of the SAD and NFF, their distribution should be assessed jointly, as they provide complementary information. In the following lines, a few examples are provided to interpret the contour map, focussing on patient ID01.

Figure 8 presents the distribution of CF, SAD, and NFF across six anatomical zones for the four stent-graft deployments for patient ID01 and ID02. SAD represents how much of the stent touches the aortic wall (quantity of contact), whilst CF indicates how strong the contact is (quality of contact). NFF, derived from both SAD and CF, reflects the overall resisting frictional force that contributes to anchoring and preventing device migration. Taken together, SAD, CF, and NFF provide a comprehensive picture of stent-graft fixation quality—capturing both the extent and intensity of contact, which are essential for assessing mechanical stability.

As an example, in patient ID01 (Fig. 8, top), the VC device shows higher SAD in zone 1 (0.051) than in zone 2 (0.037), suggesting more struts in contact with the aorta. However, the violin plot reveals that zone 2 has a higher median CF (0.039 N) than zone 1 (0.018 N), indicating stronger, but fewer contact points in zone 2. Considering the whole device, zone 2 consistently shows the highest median contact force (0.039 N) and a wide force range (up to 0.37 N), suggesting intense and variable interactions. In contrast, zone 6 has the most contact points (SAD 0.15) but the lowest median CF (0.022 N), pointing to broader but gentler engagement. Quantitatively, the net friction force (NFF) is highest in zone 3 (2.5 N), reflecting the cumulative effect of both contact area and force magnitude. Whilst zone 2, despite having the highest median CF, results in a lower total NFF (1.00 N) due to fewer contact points. This highlights how strong but sparse contact can contribute less to overall fixation than widespread moderate contact, emphasizing the importance of assessing both distribution and magnitude to understand anchoring performance.

## Discussion

Various SGs for treating thoracic aortic pathologies are available, each distinguished by its specific design and materials. The combination of materials and design gives each SG a different mechanical performance. Selecting the appropriate device for each patient is crucial for the TEVAR procedure success and for minimizing complications. Therefore, to enhance our understanding of SG performance, studying stent-graft mechanical behaviour and their interaction with aortic anatomies is essential.

In recent years, some research groups used in silico methods to evaluate the mechanical performance of SG, both under idealised testing conditions and when implanted in patient-specific aortic anatomies [10–13, 15]. However, it is important to recognize that predicting post-procedural outcomes using numerical models requires high-fidelity simulations and rigorous validation to ensure model reliability. To the best of our knowledge, no previous TEVAR numerical simulations of commercial SG models have been validated, nor have any studies compared the mechanical behaviour of SG within patient-specific anatomies. In our previous study [22], we addressed this gap by applying the V&V40 standard [2] to validate the Valiant Captivia (VC) SG model and its deployment simulation. On that basis, this study focuses on validating and comparing the mechanical behaviour of four commercially available SGs commonly used in TEVAR, which differ in design and material behaviour. The VC, TBS, and CZA devices consist of discrete nitinol stent struts sutured to a PET graft, whereas the CTAG stent-graft comprises a single sinusoidal nitinol wire bonded to an ePTFE graft. Due to its denser metal mesh, CTAG exhibited a higher overall metal-to-artery (MTA) ratio. However, when focussing on the first proximal cm, the MTA ratio also increased for VC and TBS due to the presence of more metal structures, specifically, a small ring in VC and overlapping rings in TBS.

Ad hoc crimping experimental tests were carried out on stent-grafts and used for device model validation. When comparing the crimping simulation to the experimental test, a close match of the two curves was achieved (R^2^ = 0.73 on average) for all devices, thus validating the SG models. Considering only the experimental curves, in general, CTAG revealed both a higher radial force (+106%) and a non-constant slope on the loading/unloading path of the curve in the working range due to the design [12].

The implantation under CT in the rigid patient-specific phantom allowed us to replicate and validate the TEVAR procedure in a controlled environment, eliminating uncertainties related to the aortic wall material properties. Numerical simulations were developed to reproduce the real SG implantation steps for each device model. The stent configurations obtained from these simulations showed a good agreement with the stent segmented from experimental tests, with opening area errors below 5%.

Following the successful validation of the numerical models and TEVAR simulations, the analysis was extended to investigate the mechanical performance of the SG in patient-specific anatomies. The main findings of different computed parameters are discussed in order to compare the influence of device design on their performance.

### G-APP

G-APP reflects the post-deployment configuration of the graft, including areas of folding, and it may be associated with the risk of proximal and/or distal Endoleak type IA and IB or the presence of kinking. G-APP was highly influenced by the device design, as can be evinced by the simulations of device deployment in a straight tube. CTAG showed higher G-APP values along the graft length compared to the others, due to its denser stent mesh, which reduced the creation of graft folding. The TBS design was similar to VC, but the presence of the lateral bar in TBS led to reaching larger G-APP in the central portion of the device. On the other side, the CZA device exhibited more pronounced folding, possibly attributed to its coarser suture pattern (6 mm spacing between suture points, compared to 1 mm in TBS and VC). Moving to patient-specific cases, all scenarios showed higher G-APPs in the proximal and distal covered regions, indicating acceptable apposition of the device to the aortic wall. As expected, lower G-APP values were observed at the level of the pathology or supra-aortic branches. CTAG showed higher average G-APP values across the covered length in both patients, for the same reason explained above. Additionally, its continuous sinusoidal stent design and shorter stent height (10 mm vs. 15 mm) may contribute to improved conformability to vessel anatomy [12]. In contrast, VC exhibited higher G-APP in the proximal landing zone, potentially due to the proximal support ring, which could facilitate graft expansion, reducing the formation of folding. The presence of the lateral bar in TBS seems to increase the bending stiffness of the device, and this translates into a decrease in G-APP in the patient-specific scenario with respect to the idealised one.

### Stress

Pre-TEVAR von Mises stress distribution was consistent across all patients, reflecting vessel prestress conditions, particularly in high-curvature regions such as the origins of the supra-aortic vessels [21]. Post-TEVAR von Mises stress distributions were influenced by stent-graft expansion. VC, TBS, and CZA display similar stress distribution, corresponding to their comparable metal stent designs. These stress patterns were especially visible in the descending aorta. CTAG, in contrast, exhibited a more uniform but higher stress distribution due to its denser wireframe. VC and CTAG both showed higher stress values in the aortic arch than TBS and CZA, likely due to their larger radial forces which increased device-wall contact. The locations of peak stress varied: for VC, TBS, and CZA, stress was concentrated in areas of narrow curvature, whilst in CTAG, higher stresses also appeared where the aortic diameter decreased moving distally from the penetrating aortic ulcer. This behaviour can be explained by the device’s force response during crimping and unloading, with higher exerted forces at smaller diameters (103.2 N @21.5 mm, 91.4 N @24 mm, 42.5 N @30 mm). In regions of high curvature, the larger MTA ratio and stent density of the CTAG SG might help distribute stress more evenly, reducing the likelihood of localized strut apposition. Localized stress concentrations can lead to increased aortic wall strain, potentially heightening the risk of wall damage in fragile aortas. In fact, high stress levels associated with significant aortic deformation can also contribute to vascular remodelling or dilation in the landing zones [6, 12].

### CF, NFF, and SAD

Contact force (CF), net friction force (NFF), and stent apposition density (SAD) are indicators of stent-graft implantation stability, reflecting the device’s resistance to motion. These metrics are influenced by patient-specific anatomy, SG design, and final deployment configuration. Generally, a higher radial force from the device corresponds to increased CF and NFF, which may reduce the risk of migration [6]. SAD measures the density of stent struts in contact with the aortic wall and complements G-APP, which assesses graft (fabric) apposition. Whilst G-APP captures how closely the fabric lies against the vessel with information on the foldings, SAD highlights the mechanical “anchoring” at the metal stent strut level. Together, SAD and NFF maps offer a more complete picture of the device–wall interaction. Based on their spatial patterns, the following scenarios may indicate critical conditions:

(I) Low SAD may suggest poor apposition, increasing the risk of migration or endoleaks. If paired with high NFF, localized wall stress may also increase the risk of injury.
(II) High SAD with low NFF may indicate good contact but insufficient friction force, potentially allowing for device slippage.
(III) High SAD and high NFF could enhance stability but may also result in excessive force exerted to the aortic wall, potentially critical in diseased anatomies.

In this context, analysing Fig. 8, TBS exhibited both SAD and NFF low in central regions of both patients, likely due to the presence of a lateral bar, which may increase bending stiffness and limit expansion in tortuous anatomies. CTAG, in the distal region, showed both high SAD and NFF, consistent with its stress distribution and radial force characteristics**.** VC and CZA presented no major critical risk patterns and generally demonstrated average moderate apposition metrics with a tendency of CZA towards low NFF and low SAD.

The analysed quantities and their possible correlation with TEVAR outcomes are reported in Table 6. When interpreted collectively, these engineering metrics offer a comprehensive picture of the device–aorta interaction and may help identify potentially favourable or critical implantation scenarios.

**Table 6.** Investigated parameters (engineering quantities) in relation to the clinical outcome

Clinical outcome	Engineering quantity	Motivation
Device deployed configuration	*G-APP*	The G-APP reflects the presence of folding and the good/bad apposition of the graft to the aortic wall
Device migration	*CF, NFF, SAD*	CF and NFF reflect the resistance of the stent-graft to motion, in relation to the SAD to the aortic wall [1]
Development of type I endoleaks	*G-APP*	As G-APP represent the configuration of the device after deployment and is related to the presence of folding and good/bad apposition, it can also be related to the probability of developing type I endoleak with blood flowing outside the device [5]
	*CF, NFF, SAD*	CF and NFF reflect the resistance of the stent-graft to motion: if the stent-graft moves, the probability of blood flowing outside the device increase [20]
Vessel wall overstress or remodelling	*Stress*	Large stress on the aortic wall may damage the vessel wall stress can also damage a friable aortic wall [5, 7]
	*CF, NFF, SAD*	CF and NFF with small and localized SAD can create localized remodelling and damages to the wall [6, 7, 14]

G-APP, graft apposition; CF, contact forces; NFF, net friction forces; SAD, stent apposition density

The combined analysis of G-APP, stress distribution, forces, and SAD may not only quantify device performance but also enables the identification of risks associated with migration, endoleaks, aortic wall lesions, and/or remodelling.

Although the small sample size limits generalizability, consistent device-specific trends were observed across the two patient-specific anatomies. In the more tortuous configuration (Patient ID01), devices with denser metallic structures such as CTAG and VC achieved higher apposition and stability, particularly in the proximal regions, although at the cost of slightly higher aortic wall stress. Conversely, TBS and CZA, characterized by sparser stent layouts, showed lower wall stress but reduced apposition, especially in highly curved areas. In the less tortuous case (Patient ID02), differences amongst devices were less pronounced, confirming that anatomical curvature and landing zone length play a key role in device performance.

## Limitations

For the first time, we have proposed parameters that could help assess the outcome of the TEVAR procedure, but this study is not free from limitations. Increasing the number of patients and devices would certainly aid in generalizing the study findings. Due to the limited patient sample with relatively similar yet challenging geometries, no absolute thresholds were defined to classify parameters such as “high” or “low”; nonetheless, the trends observed may still offer valuable insights into potential clinical outcomes. To establish a clinical correlation for the proposed quantification, long-term follow-up data as well as a larger patient population will be required. The anatomies that are presented in this manuscript may not be indicated for the treatment with the SG used in this study. In fact, it is important to notice that the selected device lengths were suitable for comparative analysis, but they may not represent the optimal clinical choice for the included anatomies. Additionally, although a more complex hyperelastic formulation was available, the aortic anatomies were modelled using a linear elastic material. This choice is justified by the comparative focus of the study. Lastly, this study only focuses on structural aspects, and the effect of blood flow on the SG is not taken into account.

## Conclusions

In the context of thoracic SG modelling, this study offers a comprehensive assessment of the mechanical performance of several commercially available devices, supported by a rigorous validation process. Our findings reveal a clear relationship between device design, material properties, and mechanical behaviour during deployment. By introducing a set of engineering metrics– including graft apposition, wall stress, contact forces, and friction forces– we demonstrate that device selection can play a crucial role in the success of the TEVAR procedure. Notably, higher metal density both in the proximal and in the central regions tends to enhance radial force, which improves wall apposition but also increases stress on the aortic wall. In contrast, when less metal is present (i.e., separated stent struts, without lateral bar), the device produces lower radial forces, reducing wall stress but at the cost of more localised or scattered peak stress regions due to uneven load distribution. The proximal and distal stent-graft areas are those requiring more attention and are more beneficially influenced by the presence of additional or dense rings.

The simulations highlight that each SG presents peculiar advantages and limitations, with performance varying significantly depending on patient-specific anatomy. These results support the growing consensus that there is no single optimal device, but the selection should be personalized to the patient. In this context, this study marks a step towards the integration of in silico tools into pre-operative planning for TEVAR, offering quantitative insights to aid in device selection. Ultimately, the interplay between device mechanics and anatomical features appears to directly influence not only biomechanical outcomes but also potentially clinical results as well. Future work involving a broader range of anatomies and device sizes will be essential to confirm these trends and move towards the development of predictive models with clinical applicability.

## Supplementary Information

Below is the link to the electronic supplementary material.

Supplementary file1 (MP4 554 KB)

Supplementary file2 (MP4 521 KB)

## Data Availability

Data and material involved in this study can be obtained on request by contacting the corresponding author.
